# Factors Influencing the Distribution of Endemic Damselflies in Vanuatu

**DOI:** 10.3390/insects12080670

**Published:** 2021-07-26

**Authors:** Natalie A. Saxton, Erica M. Paxman, Abigail M. Dean, Colin R. Jensen, Gareth S. Powell, Seth M. Bybee

**Affiliations:** 1 Department of Biology, 4102 LSB, Provo, UT 84602, USA; ericapaxman@gmail.com (E.M.P.); abigail.lua.dean@gmail.com (A.M.D.); colinjensen333@gmail.com (C.R.J.); garethpowell@byu.edu (G.S.P.); seth.bybee@byu.edu (S.M.B.); 2 Monte L. Bean Museum, Brigham Young University, Provo, UT 84602, USA

**Keywords:** damselfly, Vanuatu, niche model, pH, freshwater

## Abstract

**Simple Summary:**

Predicting the distribution of endemic insects is vital to continual study and conservation efforts. Here we used ecological niche models and pH data to determine which environmental factors may be influencing the distribution of a group of damselflies in Vanuatu. We tested the utility of niche models in this context and found pH to be a strong predictor for this genus.

**Abstract:**

*Vanuatubasis* Ober and Staniczek is a genus of damselfly endemic to Vanuatu. Little is known about the distribution and general natural history of the genus. We present the results of 14 weeks of fieldwork in Vanuatu to provide a better understanding of the biology of this genus. Specifically, we tested ecological niche models to predict the presence of *Vanuatubasis* throughout the region and explored how water pH may play a role in their distribution and ecology. The results of this fieldwork refined our model and further predicted the presence of this genus on additional islands. We also found stream pH as a strong predictor for the presence of *Vanuatubasis*, with their presence in alkaline streams significantly higher (*p* < 0.001). The mean pH for those streams where the genus was collected was 8.44 (*n* = 53).

## 1. Introduction

The ability to reliably predict and record the distribution of organisms is critical to both biodiversity research (e.g., taxonomy, systematics, phylogenetics) and conservation efforts [1,2]. Recording distributions is particularly important for organisms more vulnerable to extinction, such as highly endemic or rare species [3]. Freshwater ecosystems represent a fraction (~1%) of terrestrial area and are a major epicenter of animal diversity, with insects representing the majority of aquatic diversity (~80%; [4]). Island fresh-water habitats are among the most unique and threatened ecosystems on earth [5]. Island organisms are often highly unique and inhabit small areas, making them more prone to extinction [6,7]. Thus, endemic aquatic insects in freshwater island habitats are potentially the animals most at risk of disappearing before they can be properly identified by science. Documentation of the natural history, distribution, and diversity of such species, or even groups of closely related species with similar ecologies, should be an urgent focus of biodiversity research. 

Vanuatu is a relatively young island chain (~14 Ma) composed of ~82 islands, mostly volcanic, located on the convergent boundary between the Australian and Pacific plates [8]. Many of these islands have little infrastructure and are largely covered with primary and secondary rainforest, streams, lakes, and active volcanoes [9]. The islands are relatively small, with the largest being ~3955 km^2^ and over fourteen islands being less than 100 km^2^ [9]. Although there is a relatively large amount of underdeveloped area [10] compared to other island nations in the South Pacific, the islands of Vanuatu are changing rapidly. Deforestation, overgrazing, mining, and globalization are all immediate factors that contribute to land degradation and destruction of natural land cover [11,12]. Rising sea levels also threaten the limited suitable habitat for geographically restricted island endemics [13].

*Vanuatubasis* is an endemic damselfly genus in the family Coenagrionidae. Little is known about their natural history, distribution, diversity, and ecological tolerances. Currently, this genus is composed of three species described from the islands of Aneityum, Espiritu Santo, and Malekula [14]. Additional work suggests there are several new species awaiting formal description [10]. All species described and awaiting formal description appear to be endemic to a single island [15]. All species appear to share similar ecologies, yet the genus lacks documentation of ecological factors that could predict their distribution.

Ecological factors influencing the distribution of *Vanuatubasis* throughout Vanuatu are unclear. Predictive models can help to investigate some of these potential factors. Species distribution modeling tools are common and widely used in ecology, and provide insight into species’ distributions, including odonates [16,17,18,19,20,21]. These models determine relationships between occurrences of species and environmental factors at given locations and predict suitable habitat distributions based on these factors [22]. Studies have shown that species distribution models are useful for guiding field surveys toward regions where there is an increased probability of finding new populations of a known species [23,24,25]. These models are especially useful to gain a basic understanding of rare and understudied groups [26], such as *Vanuatubasis*. These models have also been shown to be accurate across a genus when the species are closely related and tend to inhabit similar ecological niches. This shared niche space between closely related species is known as ‘phylogenetic niche conservatism’ and is seen in many different taxa [27,28,29].

By increasing the discovery rate of new populations, conservation planning in poorly known and highly threatened landscapes will become more efficient [30]. Ecological niche models are a tool that can be used to more efficiently direct efforts to discover these populations, particularly in groups with limited ranges in areas that are logistically problematic to survey [20,31]. Often these models are implemented in programs such as MaxEnt [32]. Several recent studies utilize this program in various animal groups around the world. For example, Patten et al. [33] discuss the predictive power of adult presence on odonate breeding across Oklahoma. These methods have also been used to predict the effects of climate change on high-elevation insects in Italy [34].

Ecological niche models, however, are limited by which environmental layers are provided by the user [35]. Thus, potentially important ecological factors that are not able to be included in these data layers will be ignored [30]. This may be impactful for species whose distribution is largely limited by abiotic characteristics of freshwater habitats whose data are not available in certain parts of the world. Factors that are known to influence odonate distributions include sunlight availability [36,37], water turbidity [38,39], substrate type [40], vegetation [41], and the water pH [42,43]. In regard to *Vanuatubasis*, prior observations seemed to show that streams where the genus was collected had large mineral deposits, likely a result of high amounts of calcium carbonate, which can lead to basic pH levels [44]. Thus, water pH may be an important factor influencing *Vanuatubasis* distribution that is not directly accounted for in the MaxEnt model.

Here, we provide a comprehensive survey of *Vanuatubasis* resulting from 14 weeks of fieldwork spanning three years. We provide an initial ecological niche model for the group using data from 2006, 2017, and 2018, which we were able to subsequently test, validate, and improve after additional fieldwork in 2019. We also explore the relationship between the distribution of this genus and the pH levels of streams in order to explore whether *Vanuatubasis* requires alkaline environments. 

## 2. Materials and Methods

### 2.1. Fieldwork

**Figure 1:**
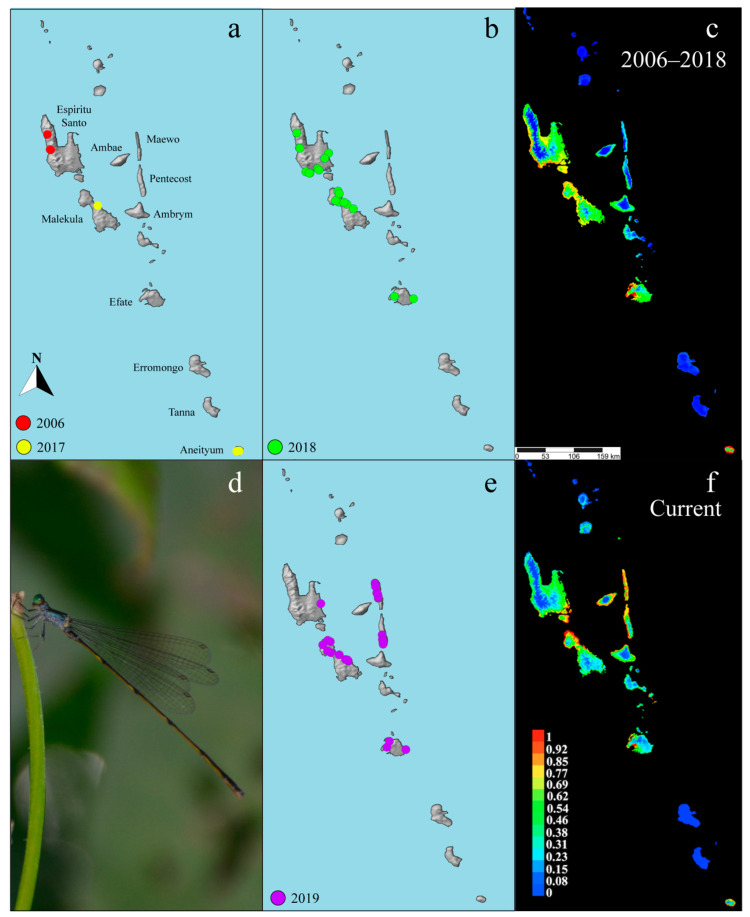
(**a**) Collecting localities pre-2018 used in the first model including data from 2006 and 2017; (**b**) Collecting localities from 2018 used in the first model; (**c**) First ecological niche model produced in MaxEnt using data from 2006–2018; (**d**) undescribed male *Vanuatubasis* from Efate; (**e**) collecting localities added to the model from 2019 fieldwork; (**f**) updated ecological niche model produced in MaxEnt with all localities.

In May and June of 2018, our research team traveled to Vanuatu, targeting the endemic genus, *Vanuatubasis*. We visited the islands of Efate, Erromango, Espiritu Santo, Gaua, Malekula, and Tanna (Figure 1b). Adult specimens were collected with aerial nets and preserved in 95% EtOH. Photographs were taken of these behaviors, and vouchers were collected. The GPS coordinates from any successful collecting event were combined with the coordinates taken during previous research expeditions in 2006 and 2017 [10,14] (Figure 1a) and used to generate a preliminary ecological niche model to guide future collecting efforts. 

In order to test the accuracy of the 2018 ecological niche model, a follow-up expedition returned to Vanuatu in May and June of 2019 to collect on the islands of Ambrym, Efate, Espiritu Santo, Maewo, Malekula, Pentecost, and Tanna (Figure 1e). The 2018 model was used as a guide for choosing sampling areas based off of their predicted habitat suitability. Three previously unstudied islands, predicted to have habitat suitable for *Vanuatubasis* were included (i.e., Ambrym, Maewo, and Pentecost). Additionally, we expanded our research areas on the islands previously studied to further validate and refine the model. 

### 2.2. Ecological Niche Models

The 2019 expedition was directed by the results of the 2018 model. Methods used to generate the first (2018) ecological niche model for *Vanuatubasis* included data points from expeditions prior to 2019 (Figure 1a,b). The updated ecological niche model generated after 2019 used all previous data and data newly collected in 2019 (Figure 1e) (See Table S1). Both ecological niche models were generated using the maximum entropy modeling software MaxEnt v3.4.1 [32] and followed basic methods discussed by Zaspel et al. [45]. MaxEnt uses species presence data and environmental variables to generate predictions and was chosen because it performs well for predictions of presence-only data [32,46]. 

We acquired environmental variables (i.e., climate data layers) from WorldClim v1.4 Current Conditions at the spatial resolution of 30 arcseconds [47]. Data layers included all 19 available bioclimatic variables and an additional four temperature and precipitation layers (e.g., monthly average temperatures (minimum, maximum, and median), monthly average precipitation, etc.). We did not reduce any of our layers since Elith et al. [19] states “MaxEnt has an inbuilt method for regularization (L1-regularization) that is reliable and known to perform well [48]. It implicitly deals with feature selection (relegating some coefficients to zero) and is unlikely to be improved—and more likely, degraded—by procedures that use other modelling methods to pre-select variables (e.g., [49]).” Each data layer was imported into DIVA-GIS v7.5 [50] and trimmed to only include Vanuatu (using bounding box coordinates: x = −13.07580, −20.34111 and y = 166.43738, 170.01268). Trimmed climate data files and all *Vanuatubasis* decimal degree localities were imported into MaxEnt [32]. We employed the jackknife method to measure variable importance. The model was generated with the default feature classes (linear, quadratic, product, and hinge) and a Cloglog output, as Cloglog is more robust with smaller data sets [51]. The model was also run with the experimental setting to write background predictions. An additional jackknife method was used to measure the importance of contributing variables. Each prediction point is given a value ranging from 0 to 1.0, with larger numbers representing a higher probability of species presence. 

### 2.3. Stream PH

In 2019, we sampled 53 streams. Fieldwork was conducted during the sunniest part of the day, between 09:00 and 14:00 h. At each surveyed location we recorded the pH using a High Accuracy pH meter (Dr. Meter, Union City, CA, USA). We recorded several points throughout the stream and the average pH was recorded. Data were only used in the final analysis if it met three criteria: (1) GPS coordinates and pH values were collected; (2) a minimum threshold of collecting time was met (>4.5 working hours); (3) the weather was favorable for odonate collecting (i.e., sunny) and wind and/or rain were not a confounding factor. In total, 42 rivers met our criteria and were included in the analysis (See Table S2). Tableau software (Salesforce, Mountain View, CA, USA) was used to depict locations surveyed and the range of pH levels across the islands. We performed an independent samples Mann–Whitney U test using SPSS Statistics v.27 (IBM, Chicago, IL, USA) to test for a significant correlation between pH-level and the presence or absence of *Vanuatubasis*. 

## 3. Results

### 3.1. 2018. Ecological Niche Model

*Vanuatubasis* were collected on the islands of Aneityum, Efate, Espiritu Santo, and Malekula at 23 different sites across these islands during 2018. *Vanuatubasis* was not found on Erromango, Gaua, or Tanna. Based on climate data associated with the confirmed collection sites, the 2018 ecological niche model predicts the presence of *Vanuatubasis* on the islands of Ambae, Ambrym, Aneityum, Efate, Espiritu Santo, Maewo, Malekula, and Pentecost with greater than 50% statistical support (Figure 1c, Table 1). The 2018 ecological niche model recovered five environmental factors that had >10% contribution to the overall model (Table 2). Here, temperature seasonality refers to the amount of temperature variation over a given year based on the standard deviation of monthly temperatures. The mean temperature of the wettest quarter is an index that approximated the mean temperature during the wettest three months of the year [52].

### 3.2. Field Validation of 2018 Model

Fieldwork in 2019 was used to validate and refine the 2018 model. In 2019, we surveyed Ambrym, Efate, Espiritu Santo, Maewo, Malekula, Pentecost, and Tanna for *Vanuatubasis*. Specimens were collected on the islands of Efate, Espiritu Santo, Malekula, Maewo and Pentecost at 31 localities (Figure 1e). The 2018 model predicted three previously unsampled islands (Ambrym, Maewo, and Pentecost) as suitable for *Vanuatubasis*. We collected *Vanuatubasis* on two of these islands (Pentecost and Maewo). No specimens were found on the islands of Ambrym or Tanna (Table 1). Ambrym was the only sampled island where *Vanuatubasis* was predicted but none were found. Ambae was also predicted to have *Vanuatubasis* present, but due to recent volcanic activity the island was not sampled.

### 3.3. 2019 Ecological Niche Model

The updated ecological niche model showed an overall refinement of the predicted distribution when compared with the 2018 model (Figure 1c,f). The probability of *Vanuatubasis* presence on the islands of Pentecost and Maewo increased, with a larger portion of the islands exhibiting probabilities close to 1.0. The probability of *Vanuatubasis* presence on the island of Malekula increased on the northern coast and shifted towards the eastern coast on Espirtu Santo. In addition, the five environmental factors that each had greater than 10% contribution to the overall model shifted so that only two of these factors were in common with the 2018 model (Table 2). Temperature seasonality and October precipitation, both in the top five for the 2018 model, increased in their contribution to the 2019 model. 

### 3.4. Stream PH

**Figure 2:**
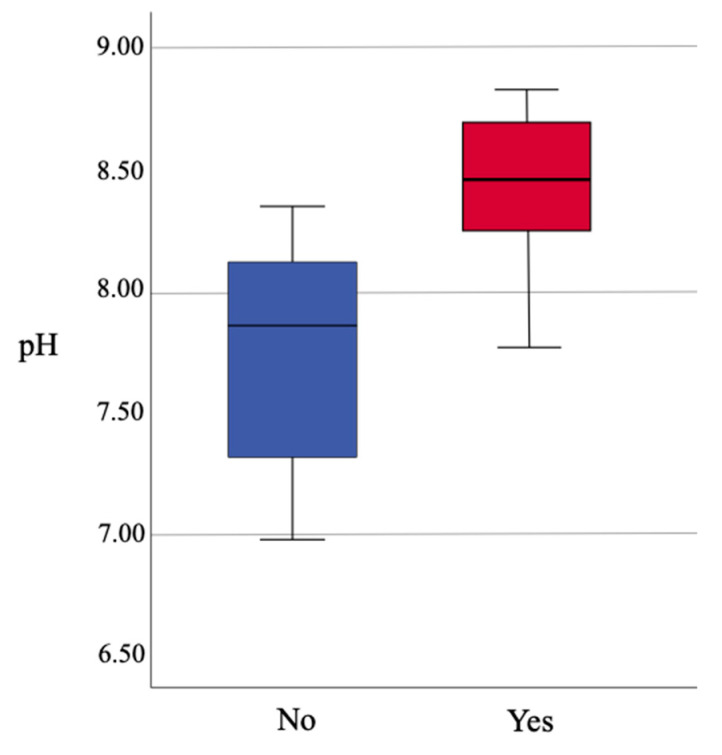
Boxplot comparing water pH to presence/absence of *Vanuatubasis*.

**Figure 3:**
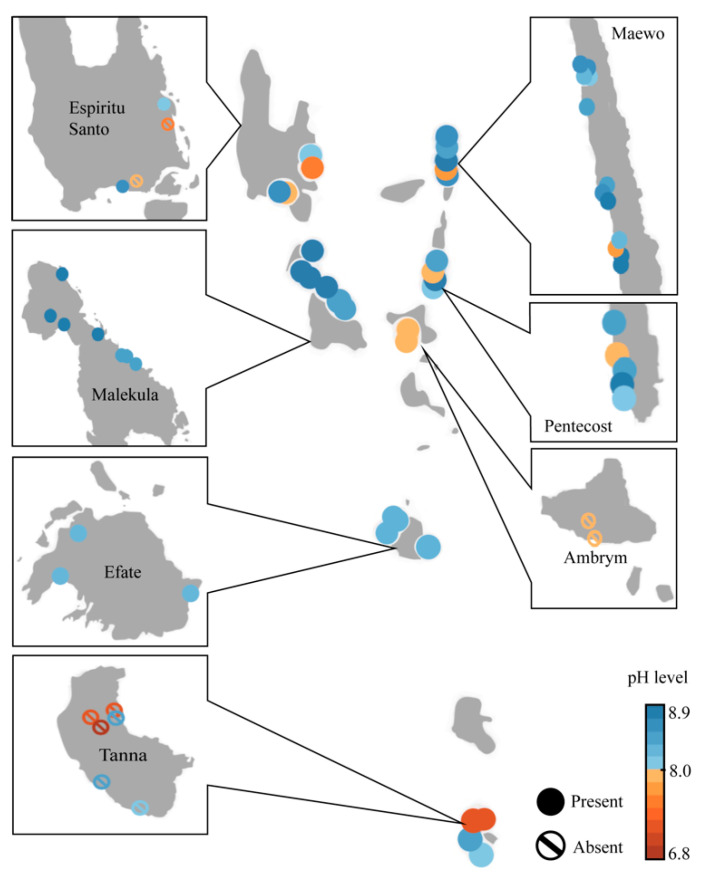
Map of Vanuatu depicting the pH of each collecting locality. Solid circles represent localities where *Vanuatubasis* was collected, and the circle-backslash symbols represent locations where *Vanuatubasis* was not found.

The pH is shown to be highly correlated with the presence of *Vanuatubasis* (*p* <0.001). The range of pH for streams where *Vanuatubasis* was collected was 7.76 to 8.82 (N = 32, mean = 8.44, SD = 0.267; Figure 2). On two occasions specimens were collected in streams with a pH < 8.0; a small population was found in a river with a pH of 7.90, and two males were found on a seep with a pH of 7.76 (Figure 3). In streams where other factors (e.g., stream bottom composition, vegetation, size and overall appearance) seemed favorable but *Vanuatubasis* was absent, the mean pH was 7.76 and the median was 7.85. (N = 10, mean = 7.76, SD = 0.467). As pH is treated on a logarithmic scale the difference between the means of streams with *Vanautubasis* present and those without is approximately 7 times [53].

## 4. Discussion

*Vanuatubasis* is only beginning to be understood in terms of its diversity, distribution, and natural history [10,14,15]. All species of *Vanuatubasis* are single island endemics, often found on a small portion of a single island. Therefore, most species within the genus are vulnerable to habitat and climate change. Ecologically, it appears that the genus *Vanuatubasis* displays a very narrow niche compared to other endemic genera of the South Pacific such as *Nesobasis* Selys and *Megalagrion* McLachlan [54,55]. *Vanuatubasis* inhabit nearly identical streams across the islands sampled here and appear to be highly endemic [15]. While some variation exists in the habitats *Vanuatubasis* occupies, they are generally found in lotic streams ranging from 7–10 m wide and 0.2–0.5 m deep. The banks of the streams tend to be sloped and densely covered with vegetation. Adult *Vanuatubasis* are frequently found in shady sites where sunspots peak through the canopy overhead, perched on vegetation, particularly ferns. The streams also appear to have high mineral content, often with visible dams formed from mineral deposits laid down over submerged mud, rocks, and branches [10]. This ecological specialization is not as pronounced in other island genera such as the Hawaiian *Megalagrion* that develop as nymphs in a wide range of habitats including seeps, pools, and leaf litter [55] allowing multiple species to be present on the same stream.

Future conservation efforts for these narrow endemics are reliant on studies focused on their distribution, as well as the environmental factors influencing this distribution. These studies will allow for better field surveys and management of their populations [35,56]. Improving our understanding of this genus, including more careful observation, sampling and taxonomy, will allow us to explore their origins, speciation, and general evolution. 

### 4.1. Ecological Niche Models

The use of ecological niche models for *Vanuatubasis* across Vanuatu proved useful to guide fieldwork efforts. Of the eight main islands on which *Vanuatubasis* was predicted to be present, we were able to find specimens on six (Table 1). We performed fieldwork in 2017–2019. Ambae was inaccessible in 2017 due to cyclone Donna and in both 2018 and 2019 due to severe volcanic activity and subsequent evacuations of the entire island. Despite this, to date none of the other islands where we performed fieldwork that had active volcanoes had *Vanuatubasis* populations. Major volcanoes can be found across the central chain of islands in Vanua Lava, Gaua, Ambae, Ambrym, and Tanna [57]. For example, Ambrym was moderately predicted to have *Vanuatubasis*; however, despite being in what we considered suitable habitat, our field team did not find *Vanuatubasis* on this island. Ambrym had a limited number of accessible streams on the southwest portion of the island, the area most strongly predicted for *Vanuatubasis* for the 2006–2018 model, and also had relatively poor weather while fieldwork was conducted. Both factors could have impacted our ability to collect *Vanuatubasis* on Ambrym. 

Fieldwork in 2019 also refined our model by increasing the probability of their presence on Ambae, Maewo, Pentecost, and Ambrym. This increase is likely due to additional presence points added, particularly in Maewo and Pentecost. Interestingly, the top five environmental factors contributing to the 2019 model only overlapped in two factors of the 2018 model (Table 2). This model was developed at a generic level due to both the lack of taxonomic work on the group as well as what appears to be very similar ecological niches among all species. Producing species-level ecological niche models in the future may provide more insight into whether certain factors are more significant to specific species. The variables that are used in niche modeling are based on the physical environment, but there may be other biotic and abiotic factors that are important to the distribution of these organisms, that are not represented by the model [30]. As mentioned previously, active volcanoes and the subsequent factors accompanying them (e.g., weather patterns that volcanoes can generate and/or the effect they can have on the water in surrounding rivers and streams) may not be accounted for in the model. Another factor that we observed and have statistical support for, but was not included in our ecological niche model, was water pH. 

### 4.2. Water PH

Water pH is notable as we were able to demonstrate a clear link between *Vanuatubasis* and alkaline streams. Studies looking at tolerance of water acidity among odonates have found that most groups can tolerate a wide range of pH levels [42,58]. Many studies attribute this wide pH tolerance to the broad distribution of odonates around the world [42]. Studies focusing on conservation of dragonfly groups have found that these species may even experience a population increase in response to acidification due to decreased competition and an increase in resource availability [58,59]. The general trend of tolerance to a wide range of pH has been explored across a limited set of odonates (i.e., common and easily identifiable species). The pH preferences and tolerance of more inconspicuous specialized and/or endemic odonate species are not as well known. It is likely that by studying such species, odonate distribution patterns, as they relate to water pH levels, will become clearer.

We used presence/absence data in conjunction with averaged pH measurements of streams to demonstrate that *Vanuatubasis* species are statistically more likely to be found in alkaline streams (*p* < 0.001). If pH is playing a role in the distribution of *Vanuatubasis*, it is probably most impactful on the immature, aquatic life-stage. Water pH has been recorded to affect nymphs in the past. For example, Correa et al. [60] found that in *Somatochlora cingulata* Selys, as they decreased the water pH, the respiration rate of the nymphs also decreased, particularly in the earlier stages of development. Odonate nymphs tend to live in much higher densities with both conspecifics and other congeners than adults and are often the primary source of competition for food resources [61]. This high rate of competition may force organisms to shift towards “extreme” habitats in order to limit competition [62]. Several instances of habitat shifts to avoid interspecific competition have been recorded in organisms including fish [63], lizards [64], and beetles [65]. 

Though the correlation between pH level and presence of *Vanuatubasis* is strong, it is difficult to determine if the pH level is a direct causal factor (e.g., egg survivorship, larval respiration) [42], or if a basic pH affects another biotic factor (e.g., predator and/or prey presence, plant communities, accumulation/decomposition of organic matter) that in turn impacts the presence of *Vanuatubasis*. Since pH can vary seasonally [66], studies would need to be done during months with higher precipitation and higher temperatures in Vanuatu to see how these results vary or persist. Our understanding of this phenomenon would improve with a broader sampling across additional islands at different elevations.

## 5. Conclusions

*Vanuatubasis* is an evolutionarily fascinating lineage. They exhibit extreme island endemism, selective habitat preference, and interesting behaviors. Our results provide compelling evidence supporting pH as a limiting factor impacting *Vanautubasis* distributions, as well as the utility of ecological niche models to guide fieldwork. We can use this predictive model as well as our observations of ideal stream pH conditions to guide our search for *Vanuatubasis*, as we work to fully understand the ecology and evolution of this group.

## Figures and Tables

**Table 1:** Prediction of *Vanuatubasis* presence (50–90%) at different cut-offs above 50% for the 2018 model, compared to the results of 2019 fieldwork (Presence).

Island	50%	60%	70%	80%	90%	Presence
Ambae	✓	—	—	—	—	—
Ambrym	✓	✓	—	—	—	—
Aneityum	✓	✓	✓	✓	✓	✓
Efate	✓	✓	✓	✓	✓	✓
Espiritu Santo	✓	✓	✓	✓	✓	✓
Malekula	✓	✓	✓	✓	✓	✓
Pentecost	✓	—	—	—	—	✓
Maewo	✓	—	—	—	—	✓

**Table 2:** The five variables that contributed the most to the MaxEnt ecological niche models for 2018 and 2019.

WorldClim Factor	2018 (%)	2019 (%)
Nov. precipitation	22.3	—
Mean temperature of wettest quarter	18.7	—
Temperature seasonality	17.8	21.1
Oct. precipitation	16.0	19.9
Aug. precipitation	9.6	–
Feb. maximum temperature	—	11.5
Jan. precipitation	—	8.1
Oct. minimum temperature	—	6.9

## Data Availability

Supporting data can be found in the supplementary material accompanying this study.

## Supplementary Materials

The following are available online at https://www.mdpi.com/article/10.3390/insects12080670/s1, Table S1: Collecting localities, Table S2: pH data by locality.
