# Supplementary material for: Factors Influencing the Distribution of Endemic Damselflies in Vanuatu
Source: Insects. 2021 Jul 26;12(8):670. doi: 10.3390/insects12080670 (PMC8396446; doi:10.3390/insects12080670)
Supplement: Supplementary file 1 [file insects-12-00670-s001.zip › insects-1218396-supplementary.pdf]

Supplementary Table S1: GPS coordinates for each occurrence record of *Vanuatubasis* used in MaxEnt analyses

Pre-2017 Island	Latitude	Longitude
Santo	-15.2098	166.6771
Santo	-15.2175	166.6871
Santo	-14.9611	166.6332
Aneityum	-20.218	169.8012
Aneityum	-20.2103	169.8083
Aneityum	-20.2194	169.7733
Malekula	-16.1435	167.4671
**2018**		
Malekula	-16.107861	167.327444
Malekula	-16.083	167.275
Malekula	-16.145944	167.465333
Malekula	-16.221361	167.571611
Malekula	-15.927333	167.318083
Malekula	-15.968556	167.339667
Malekula	-16.114278	167.420389
Santo	-15.295705	167.161609
Santo	-15.5538	166.9814
Santo	-15.6275	166.853972
Santo	-15.607787	166.784943
Santo	-15.629558	166.842575
Santo	-15.382895	167.095869
Santo	-15.57815	167.002433
Efate	-17.675229	168.254133
Efate	-17.42.840	168.34.129
**2019**		
Efate	-17.7142	168.5688
Malekula	-16.1079	167.3273

Malekula	-16.1435	167.4671
Malekula	-15.9256	167.3192
Malekula	-15.904	167.2772
Malekula	-15.9803	167.1928
Malekula	-16.2208	167.5723
Malekula	-16.2475	167.6168
Malekula	-16.2292	167.6034
Malekula	-16.0787	167.2726
Maewo	-15.1263	168.0937
Maewo	-15.1191	168.0891
Maewo	-15.1178	168.0871
Santo	-15.2955	167.1614
Maewo	-15.2067	168.112
Maewo	-15.2034	168.1132
Maewo	-15.198	168.1138
Maewo	-15.1901	168.1107
Maewo	-15.1811	168.1137
Maewo	-14.9547	168.0596
Maewo	-14.96	168.0614
Maewo	-14.9611	168.0614
Maewo	-14.9628	168.0604
Maewo	-14.9626	168.0605
Maewo	-14.965	168.0609
Maewo	-14.9654	168.0604
Maewo	-15.113	168.0926
Maewo	-15.0119	168.0667
Pentecost	-15.931	168.1897
Pentecost	-15.9581	168.1959
Pentecost	-15.9589	168.1948
Pentecost	-15.9685	168.1922
Pentecost	-15.8126	168.177
Pentecost	-15.8886	168.1803
Pentecost	-15.9088	168.1904
Efate	-17.6754	168.2559
Efate	-17.5768	168.2959

Supplementary Table S2: GPS coordinates with corresponding pH data

Island	Latitude	Longitude	pH	Presence
Efate	-17.7142	168.5688	8.24	Yes
Malekula	-16.1079	167.3273	8.72	Yes
Malekula	-16.1435	167.4671	8.70	Yes
Malekula	-15.9256	167.3192	8.77	Yes
Malekula	-16.2208	167.5723	8.40	Yes
Malekula	16.2475	167.6168	8.34	Yes
Malekula	-16.2215	167.5781	8.45	Yes
Malekula	-16.0787	167.2726	8.78	Yes
Ambrym	-16.2991	168.0877	7.86	No
Ambrym	-16.3533	168.0944	7.84	No
Santo	15.5833	167.0456	7.94	No
Santo	-15.5781	167.0022	8.51	Yes
Santo	-15.3704	167.185	7.67	No
Santo	-15.2955	167.1614	8.07	Yes
Maewo	-15.1263	168.0937	8.82	Yes
Maewo	-15.1191	168.0891	8.75	Yes
Maewo	-15.2034	168.1132	8.75	Yes
Maewo	-15.1980	168.1138	8.70	Yes
Maewo	-15.1901	168.1107	7.76	Yes
Maewo	-15.1811	168.1137	8.20	Yes
Maewo	-14.9547	168.0596	8.62	Yes
Maewo	-14.9600	168.0614	8.36	Yes
Maewo	-14.9611	168.0614	8.52	Yes
Maewo	-14.9626	168.0605	8.52	Yes
Maewo	-14.9650	168.0609	8.19	Yes
Maewo	-14.9654	168.0604	8.32	Yes
Maewo	-15.1130	168.0926	8.45	Yes
Maewo	-15.0119	168.0667	8.39	Yes
Pentecost	-15.9310	168.1897	8.67	Yes
Pentecost	-15.9581	168.1959	8.62	Yes
Pentecost	-15.9589	168.1948	8.10	Yes
Pentecost	-15.8126	168.177	8.49	Yes
Pentecost	-15.8886	168.1803	7.90	Yes
Pentecost	-15.9088	168.1904	8.41	Yes
Tanna	-19.4318	169.295	7.21	No
Tanna	-19.4489	169.3082	6.97	No
Tanna	-19.4245	169.3499	8.33	No

Tanna	-19.4179	169.3484	7.31	No
Tanna	-19.5798	169.3101	8.34	No
Tanna	-19.6389	169.391	8.11	No
Efate	-17.6754	168.2559	8.24	Yes
Efate	-17.5768	168.2959	8.19	Yes
